# Supplementary material for: Empirical methods for controlling false positives and estimating confidence in ChIP-Seq peaks
Source: BMC Bioinformatics. 2008 Dec 5;9:523. doi: 10.1186/1471-2105-9-523 (PMC2628906; doi:10.1186/1471-2105-9-523)
Supplement: Additional File 1 — A variety of html documents from the USeq web site detailing the available applications, their best usage, output file type descriptions, command line menus, etc. [file 1471-2105-9-523-S1.zip › USeqUserGuides/JavaDoc.zip!/JavaDoc/util/bio/annotation/Coordinate.html]

Coordinate


|  |  |  |
| --- | --- | --- |
| **Overview** \| **Package** \| **Class** \| **Use** \| **Tree** \| **Deprecated** \| **Index** \| **Help** | |  |
| PREV CLASS   **NEXT CLASS** | **FRAMES**    **NO FRAMES**     **All Classes** |  |
| SUMMARY: NESTED \| FIELD \| CONSTR \| METHOD | DETAIL: FIELD \| CONSTR \| METHOD |  |


---


## util.bio.annotation Class Coordinate

```
java.lang.Object
  util.bio.annotation.Coordinate
```

**All Implemented Interfaces:**: java.lang.Comparable

**Direct Known Subclasses:**: ScoredCoordinate

---

``` public class Coordinate extends java.lang.Object implements java.lang.Comparable ```

Base class for holding info about a genomic coordinate.

---

**Constructor Summary**

`Coordinate(java.lang.String chromosome,
int start,
int stop)`


| **Method Summary** | |
| --- | --- |
| `int` | `bpsIntersection(Coordinate other)`             Returns -1 for no overlap, 0 for complete overlap, or a positive int for the # bases of overlap. |
| `int` | `compareTo(java.lang.Object other)`             Sorts by chromsome, start position, length (smallest to largest). |
| `static int` | `findLastBase(Coordinate[] chromSpecific)`             Finds the last base. |
| `java.lang.String` | `getChromosome()` |
| `int` | `getStart()` |
| `int` | `getStop()` |
| `boolean` | `intersects(Coordinate other)`             Assumes coordinates are inclusive. |
| `boolean` | `intersects(int beginning, int end)`             Assumes coordinates are inclusive. |
| `static Coordinate[]` | `parseFile(java.io.File picksFile, int subStart, int subEnd)`             Parses a tab delimited chr,start,stop file. |
| `static Coordinate[]` | `readBinary(java.io.File file)`             Reads a binary Coordinate[] file. |
| `void` | `setChromosome(java.lang.String chromosome)` |
| `void` | `setStart(int start)` |
| `void` | `setStop(int stop)` |
| `static java.util.HashMap<java.lang.String,Coordinate[]>` | `splitByChromosome(Coordinate[] sortedCoordinates)`             Split by chromosome into a HashMap of chromosome:Coordinate[]. |
| `static Coordinate[]` | `subtract(Coordinate forReduction, Coordinate mask)`             Clips the forReduction based on the mask. |
| `java.lang.String` | `toString()` |
| `static boolean` | `writeBinary(Coordinate[] c, java.io.File file)`             Writes a binary Coordinate[]. |

**Methods inherited from class java.lang.Object**

`equals, getClass, hashCode, notify, notifyAll, wait, wait, wait`

**Constructor Detail**

### Coordinate

```
public Coordinate(java.lang.String chromosome,
                  int start,
                  int stop)
```


**Method Detail**

### compareTo

```
public int compareTo(java.lang.Object other)
```

:   Sorts by chromsome, start position, length (smallest to largest).

    :   **Specified by:**: `compareTo` in interface `java.lang.Comparable`

---


### toString

```
public java.lang.String toString()
```

:   **Overrides:**: `toString` in class `java.lang.Object`

---


### intersects

```
public boolean intersects(int beginning,
                          int end)
```

:   Assumes coordinates are inclusive.

---


### intersects

```
public boolean intersects(Coordinate other)
```

:   Assumes coordinates are inclusive.

---


### bpsIntersection

```
public int bpsIntersection(Coordinate other)
```

:   Returns -1 for no overlap, 0 for complete overlap, or a positive int for the # bases of overlap.
    Assumes coordinates are inclusive.

---


### subtract

```
public static Coordinate[] subtract(Coordinate forReduction,
                                    Coordinate mask)
```

:   Clips the forReduction based on the mask. Will return null if the mask completely covers the forReduction.

---


### findLastBase

```
public static int findLastBase(Coordinate[] chromSpecific)
```

:   Finds the last base.

---


### parseFile

```
public static Coordinate[] parseFile(java.io.File picksFile,
                                     int subStart,
                                     int subEnd)
```

:   Parses a tab delimited chr,start,stop file.

    :   **Parameters:**: `subStart` - - bases to be subtracted from region starts: `subEnd` - - bases to be subtracted from region ends

---


### splitByChromosome

```
public static java.util.HashMap<java.lang.String,Coordinate[]> splitByChromosome(Coordinate[] sortedCoordinates)
```

:   Split by chromosome into a HashMap of chromosome:Coordinate[].
    Don't forget to sort!

---


### writeBinary

```
public static boolean writeBinary(Coordinate[] c,
                                  java.io.File file)
```

:   Writes a binary Coordinate[].

    :   **Returns:**: true if sucessful, false if something bad happened.

---


### readBinary

```
public static Coordinate[] readBinary(java.io.File file)
```

:   Reads a binary Coordinate[] file.

    :   **Returns:**: null if something bad happened.

---


### getChromosome

```
public java.lang.String getChromosome()
```

---


### setChromosome

```
public void setChromosome(java.lang.String chromosome)
```

---


### getStart

```
public int getStart()
```

---


### setStart

```
public void setStart(int start)
```

---


### getStop

```
public int getStop()
```

---


### setStop

```
public void setStop(int stop)
```


---


|  |  |  |
| --- | --- | --- |
| **Overview** \| **Package** \| **Class** \| **Use** \| **Tree** \| **Deprecated** \| **Index** \| **Help** | |  |
| PREV CLASS   **NEXT CLASS** | **FRAMES**    **NO FRAMES**     **All Classes** |  |
| SUMMARY: NESTED \| FIELD \| CONSTR \| METHOD | DETAIL: FIELD \| CONSTR \| METHOD |  |


---
